# Monitoring antigenic protein integrity during glycoconjugate vaccine synthesis using capillary electrophoresis-mass spectrometry

**DOI:** 10.1007/s00216-016-9723-5

**Published:** 2016-07-02

**Authors:** Sara Tengattini, Elena Domínguez-Vega, Caterina Temporini, Marco Terreni, Govert W. Somsen

**Affiliations:** 1 Department of Drug Sciences, University of Pavia, via Taramelli 12, 27100 Pavia, Italy; 2 Division of BioAnalytical Chemistry, Department of Chemistry and Pharmaceutical Sciences, Vrije Universiteit Amsterdam, de Boelelaan 1085, 1081 HV Amsterdam, The Netherlands

**Keywords:** CE-MS, Intact protein analysis, Protein characterization, Neo-glycoproteins, Antigenic proteins, Glycoconjugate vaccines

## Abstract

A capillary electrophoresis-mass spectrometry (CE-MS) method was developed for the characterization and integrity assessment of the *Mycobacterium tuberculosis* (MTB) antigens TB10.4 and Ag85B and their chemically produced glycoconjugates, which are glycovaccine candidates against tuberculosis (TB). In order to prevent protein adsorption to the inner capillary wall and to achieve efficient separation of the antigen proteoforms, a polyionic multilayer coating of polybrene-dextran sulfate-polybrene (PB-DS-PB) was used in combination with 1.5 M acetic acid as background electrolyte (BGE). Coupling of CE to high-resolution time-of-flight MS was achieved by a coaxial interface employing a sheath liquid of isopropanol-water (50:50, *v*/*v*) containing 0.1 % formic acid. The MTB antigens were exposed to experimental conditions used for chemical glycosylation (but no activated saccharide was added) in order to investigate their stability during glycovaccine production. CE-MS analysis revealed the presence of several closely related degradation products, including truncated, oxidized and conformational variants, which were assigned by accurate mass. Analysis of synthesized mannose conjugates of TB10.4 and Ag85B allowed the determination of the glycoform composition of the *neo*-glycoproteins next to the characterization of degradation products which were shown to be partly glycoconjugated. Moreover, the selectivity of CE-MS allowed specific detection of deamidated species (protein mass change of 1.0 Da only), indicating that chemical glycosylation increased susceptibility to deamidation. Overall, the results show that CE-MS represents a useful analytical tool for the detailed characterization and optimization of *neo*-glycoconjugate products.

**Electronic supplementary material:**

The online version of this article (doi:10.1007/s00216-016-9723-5) contains supplementary material, which is available to authorized users.

**Graphical Abstract:**
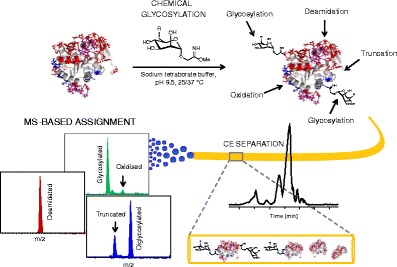
Flowchart illustrating *Mycobacterium tuberculosis* (MTB) antigen glycosylation, glycoconjugate variant and degradation product separation by capillary electrophoresis (CE) and their characterization by intact mass spectrometry (MS)

## Introduction

Prevention of infectious diseases by vaccination is considered one of the most successful health treatments. Glycoconjugate vaccines, in which oligosaccharides are conjugated to carrier proteins (*neo-*glycoproteins), are among the safest and most efficacious vaccines available [1–3]. Since the introduction of the first glycoconjugate vaccine against *Haemophilus influenzae* type b (Hib) in 1987, this strategy has been successfully applied against numerous bacterial-infectious diseases [4].

Tuberculosis (TB) still remains one of the world’s deadliest human diseases, ranked second only after immune deficiency syndrome (AIDS) in the number of people killed every year. In 2014, about 9.0 million people developed TB and 1.5 million died [5, 6]. Even though TB is curable, the efficacy of available chemotherapies is limited due to emerging multidrug-resistant (MDR) and extensively drug-resistant (XDR) strains of *Mycobacterium tuberculosis* (MTB) [5]. Therapeutic vaccination against MTB has potential for the treatment of MDR- and XDR-TB [5]. At present, *Bacillus Calmette-Guérin* (BCG), the only TB vaccine in use, has major limitations: BCG protects only infants and [7] its efficacy wanes significantly over a period of 10–15 years [8]. Thus, the design of an effective vaccine against TB remains an international research priority [6]. In this context, conjugation of antigenic proteins from MTB with arabino-mannan polysaccharides was considered for the development of highly immunogenic glycoconjugate vaccines [9].

Glycoconjugate vaccines are produced by chemical binding of oligosaccharides to carrier proteins forming *neo-*glycoproteins [10]. For this purpose, glycans are activated targeting nucleophilic groups of amino acid residues (lysines, aspartic/glutamic acids or cysteines) of antigen proteins [11]. One of the most diffused and consolidated methods implies the use of 2-iminomethoxyethyl thioglycosides (IME) that selectively react with ε-amino groups of lysine residues [12]. These glycosylation strategies, however, commonly lead to a mixture of *neo*-glycoproteins with different saccharide loading numbers and/or saccharide positionings, which may have different properties such as clearance kinetics and/or antigenicity [11]. Moreover, isolated or recombinantly produced carrier proteins, such as antigenic proteins, may not be entirely pure and contain degradation products. In addition, these proteins may undergo unwanted chemical modifications during the glycovaccine production process. Characterization of heterogeneous *neo*-glycoprotein products evidently is a fundamental, but challenging, task requiring not only determination of the generated glycoforms but also characterization of potential degradation products formed during the conjugation process in order to ensure the production of pure and stable products during the glycovaccine synthesis.

Mass spectrometry (MS)-based approaches are widely used for the structural characterization of novel glycoconjugates [13, 14]. Bottom-up approaches in which proteins are first digested into fragments, i.e. (glyco)peptides, are mainly used for the localization of glycosylation sites of novel (*neo*-)glycoproteins [15, 16]. We have shown the usefulness of bottom-up liquid chromatography (LC)-MS to characterize the glycosylation reactivity of surface amino acids of antigens using different linkers (e.g. IME or homobifunctional (4-nitrophenyl ester)). This allowed selection of the optimal activation and conjugation conditions preserving the protein antigenic epitope after glycosylation [17]. Still, quality control of the intact *neo-*glycoprotein is an important issue in biopharmaceutical analysis as it provides essential information on (glycoform) composition and protein integrity which cannot be revealed by bottom-up approaches only. Moreover, intact analysis is relatively fast, requires minimum sample treatment and avoids unwanted modifications induced by enzymatic treatments [18, 19]. We have applied direct-infusion MS methods for the characterization of the glycoform composition of intact novel glycovaccines [17]. However, direct MS characterization of intact *neo*-glycoproteins can be quite challenging due to their complex (micro)heterogeneity. In addition to the inevitable heterogeneity related to the carbohydrate component (number, structure and position), one also has to consider the potential antigen heterogeneity caused by the presence of e.g. proteoforms and degradation products. In particular, distinguishing closely related variants, such deamidated forms or conformers, might not be possible with stand-alone MS. Therefore, separation of intact *neo*-glycoprotein variants prior to MS detection is needed in order to reduce the complexity of mass spectra and will facilitate the characterization of all present species, including minor components. Conventional reversed-phase liquid chromatography (RPLC) of intact glycoproteins may be troublesome due to adverse interactions with the hydrophobic stationary phase, leading to poor performance. Moreover, RPLC often lacks the selectivity and efficiency to assess subtle protein modifications and proteoforms. Recently, hydrophilic interaction liquid chromatography (HILIC) has shown promising possibilities for resolving intact glycoforms including *neo*-glycoproteins [20–22], but coupling of protein HILIC with MS still has to be demonstrated. Capillary electrophoresis (CE)-MS has proved to be a useful and powerful analytical tool for the characterization of intact proteins, glycoproteins and drug-protein conjugates, combining high protein separation efficiency with mass-selective detection [23–26]. Unwanted interactions between proteins and negatively charged silanol groups on the inner wall of fused-silica capillaries can be prevented by applying MS-compatible coatings, allowing efficient separation of proteins in a wide range of conditions [26]. CE has shown particularly useful in revealing charge heterogeneity among protein degradation products and impurities, but also has shown the ability to separate positional isomers and conformers [25, 27, 28].

In this work, we studied the potential of CE-MS for the characterization of the antigenic MTB proteins TB10.4 (11 kDa) and Ag85B (31 kDa) which were conjugated with 2-iminomethoxyethyl-mannose (Man-IME) and 2-minomethoxyethyl-mannose(1–6)mannose (Man(1–6)Man-IME). These *neo*-glycoconjugates are candidate vaccines against TB [17]. In order to investigate the stability of the MTB antigens during glycovaccine production, the proteins were analysed by the developed CE-MS method before and after exposure to experimental conditions used for chemical glycosylation, but with no activated saccharide added. Next, the *neo*-glycoproteins were analysed by CE-MS in order to determine glycoform composition as well as to monitor the presence of glycosylation-related degradation products.

## Materials and methods

### Reagents and chemicals

All chemicals were of analytical grade. Ammonium hydroxide (30 % solution), acetic acid, formic acid, isopropanol (IPA), acetonitrile (ACN), polybrene (hexadimethrine bromide, PB; average molecular weight (MW) 15,000), dextran sulfate (DS, average MW >500,000), benzamidine hydrochloride, sodium tetraborate and phosphate-buffered saline (PBS) were purchased from Sigma-Aldrich (St. Louis, MO, USA). Deionized water was obtained from a Milli-Q purification system (Millipore, Bedford, MA, USA). IME thioglycosides (Man-IME and Man(1–6)Man-IME) were prepared according to a previously reported procedure [29]. The TB10.4 and Ag85B immunogenic proteins were obtained as recombinant forms in *Escherichia coli* as reported by Piubelli et al. [8] and finally collected in 20 mM 3-(*N*-morpholino)propanesulfonic acid (MOPS) and 0.4 M NaCl, pH 7.0 at different concentrations.

### Protein glycosylation

According to the protocol previously reported [17], the glycosylation reaction was carried out in sodium tetraborate buffer, 100 mM, pH 9.5. TB10.4 and Ag85B were dissolved in the buffer in a concentration of 5.5 mg/mL, and the solution was subsequently mixed with IME-glycoside in a glycoside/protein molar ratio of 200/1. Benzamidine chloride was added to the reaction mixture in order to reduce protein digestion by residual enterokinase derived from the antigen protein production process. The reaction mixture was vortexed for 1 min and incubated for 24 h at 25 or 37 °C under continuous stirring.

### Sample preparation

*Neo*-glycoconjugate samples and antigenic proteins exposed to glycosylation conditions for stability studies were purified in order to remove reagents and/or salts. For this, the solutions were submitted to four 20-min steps of ultrafiltration at 13,000*g* and 4 °C using Amicon® Ultra filters (Millipore, Billerica, MA, USA) with a nominal molecular weight limit (NMWL) of 3 or 10 kDa and a load capacity of 500 μL. Proteins and *neo*-glycoproteins were finally collected and stored in PBS. Prior to CE-MS analysis, the buffer of proteins and their glycoconjugates were exchanged for water by two 20-min steps of ultrafiltration at 13,000*g* and 4 °C. All the samples were diluted with water to a final concentration of 1 mg/mL.

### Capillary electrophoresis

CE analyses were performed using a Beckman PA 800 plus instrument (Beckman Coulter, Brea, CA, USA). Bare fused-silica capillaries with an internal diameter of 50 μm were obtained from Polymicro Technologies (Phoenix, AZ, USA) and cut to a total length of 90 cm. Hydrodynamic sample injections were performed at 1 psi for 12 s corresponding to an injection volume of 12.5 nL and an injected protein amount of 12.5 ng. UV detection was performed at 214 nm.

For the optimized CE-MS method, the separation voltage was −25 kV, the capillary temperature 15 °C and the background electrolyte (BGE) 1.5 M acetic acid. For the PB-DS-PB-coating, the new bare fused-silica capillaries were firstly rinsed with successively 1 M NaOH and water (both 30 min at 20 psi). Subsequently, the capillaries were flushed with 10 % (*w*/*v*) PB solution (60 min at 5 psi), deionized water (30 min at 10 psi), 0.5 % (*w*/*v*) DS solution (60 min at 5 psi), deionized water (30 min at 10 psi), 10 % (*w*/*v*) PB solution (50 min at 5 psi), deionized water (30 min at 10 psi) and BGE (10 min at 20 psi). Overnight, the coated capillaries were filled with water and the tips were immersed in vials with water.

### Mass spectrometry

MS detection was performed using a maXis HD ultra-high-resolution quadrupole time-of-flight (QTOF) mass spectrometer (Rs, 80,000) (Bruker Daltonics, Bremen, Germany) equipped with an electrospray ionization (ESI) source. CE-MS coupling was performed using a sheath liquid electrospray interface from Agilent Technologies. The sheath liquid was a mixture of IPA-water (50:50, *v*/*v*) containing 0.1 % of formic acid and was delivered at a flow rate of 3 μL/min by a syringe pump from Cole-Parmer (Vernon Hill, IL, USA). The mass spectrometer was operated in positive-ion mode with an electrospray voltage of 4.5 kV. The nebulizer gas (nitrogen) pressure was 5 psi, and the dry gas flow rate and temperature was 4 L/min nitrogen and 200 °C, respectively. Quadrupole ion and collision cell energies were 5 and 8 eV, respectively. The monitored mass range was 250–4000 *m*/*z*. Data were analysed using Bruker Daltonics Data Analysis software (Compass DataAnalysis, version 3.2). Extracted-ion electropherograms (EIEs) were obtained with an extraction window of ±0.1 *m*/*z* and using the smooth option of the software (Gaussian at 1 point). Mass determinations of proteins were performed using the “Maximum entropy deconvolution” utility. Mass accuracy employed for assignments was <20 ppm. Theoretical masses were calculated from the amino acid sequence using the “Peptide mass calculator” on IonSourceMS (www.ionsource.com).

## Results and discussion

### CE-MS method development

A BGE of acetic acid was selected for the CE-MS analysis of TB10.4 and Ag85B, as this low-pH BGE has previously shown good separation and ESI performance for intact proteins [23]. TB10.4 and Ag85B have an isoelectric point (p*I*) of 4.44 and 4.77, respectively, and therefore will be positively charged when using an acidic BGE. In order to minimize adsorption of the proteins to the inner wall of the capillary, a MS-compatible non-covalent multilayer capillary coating, consisting of PB-DS-PB, was employed. Using 200 mM acetic acid as BGE, a single symmetrical peak for TB10.4 and two partially resolved peaks for Ag85B were observed in CE-UV. Increase of the acetic acid concentration to 2 M (pH 2.2) resulted in an improved resolution of the Ag85B. When using these conditions for the analysis of the Ag85B-Man sample, two additional small peaks migrating before the main peak were observed (Fig. S1 in Electronic Supplementary Material (ESM)). The influence of voltage and temperature on separation was investigated. Voltage variation between −25 and −15 kV did not provide a significant change in the separation, whereas decreasing the temperature from 25 to 15 °C allowed improving considerably the resolution of peaks observed for the Ag85B and Ag85B-Man samples. The addition of ACN (up to 20 % *v*/*v*) to the BGE was considered, but no improvement in separation was obtained. Based on the CE-UV experiments, the finally selected CE conditions comprised a PB-DS-PB-coated capillary in combination with 2 M acetic acid as BGE, a capillary temperature of 15 °C and a separation voltage of −25 kV. For coupling CE to MS, a coaxial sheath liquid interface was employed. A sheath liquid consisting of IPA-water (50:50, *v*/*v*) containing 0.1 % of formic acid was selected as it provided good sensitivity and current stability. However, the BGE of 2 M acetic acid resulted in considerable ionization suppression of the proteins. As a compromise between CE resolution and MS sensitivity, a BGE of 1.5 M acetic acid (pH 2.3) was finally selected.

### Characterization and stability evaluation of TB10.4 and Ag85B

In order to investigate the stability of TB10.4 and Ag85B during glycoconjugate synthesis, the protein antigens were analysed by CE-MS before and after exposure for 24 h to glycosylation reaction conditions (see “Protein glycosylation”), but with no activated saccharides added. TB10.4 exposure was performed at 37 °C, being the optimum glycosylation temperature [17]. For Ag85B, glycosylation yields were 100 % at both 25 and 37 °C and, therefore, both temperatures were employed in the exposure study.

#### TB10.4

**Fig. 1:**
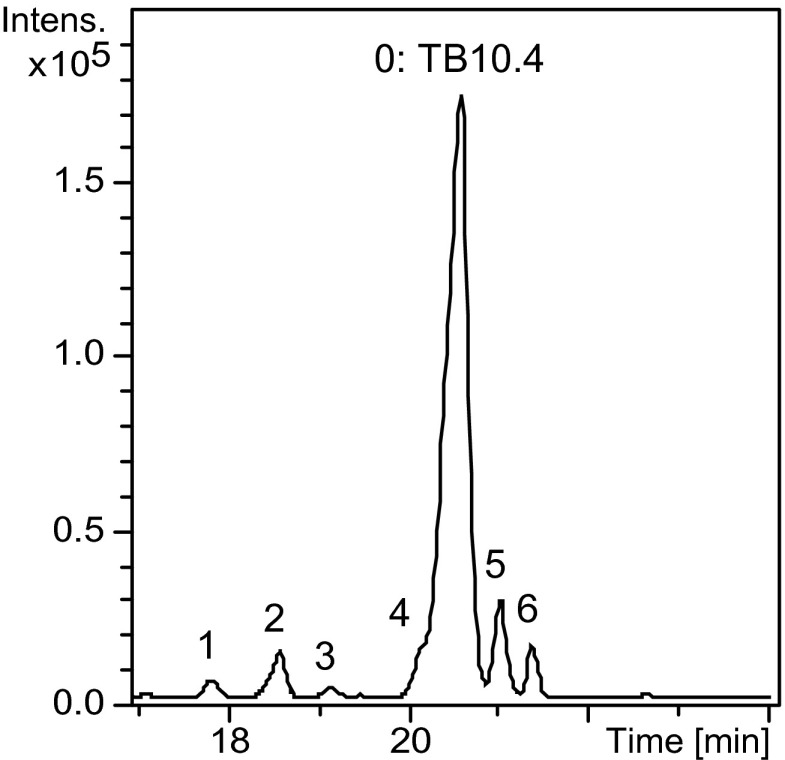
BPE obtained during CE-MS of TB10.4 which has been exposed to glycosylation conditions at 37 °C for 24 h

CE-MS analysis of TB10.4 in water showed a single symmetric peak at a migration time of approximately 20 min. Deconvolution of the mass spectrum obtained in the apex of the peak revealed a single protein molecular mass of 11,076.1 Da, which agrees well with the expected molecular mass for TB10.4 (11,076.3 Da; see ESM, Fig. S2 for the protein sequence and Fig. S3 for the deconvoluted spectrum). CE-MS analysis of the TB10.4 sample after exposure to glycosylation conditions (37 °C for 24 h) showed the presence of several degradation products (Fig. 1) which could be assigned (except peak 1) based on the masses derived from the deconvoluted spectra (Table 1). Peaks 2 and 3 were identified as composed of truncated protein forms that lost 4 to 17 amino acids from the C-terminus side: A1-N86, A1-T87, A1-M88, A1-A89, A1-M90, A1-M91, A1-A92, A1-E97 and A1-A99. The shorter migration times of these compounds with respect to TB10.4 most probably can be explained by the loss of two (R93, K100) or one (K100) basic amino acid, causing a decrease of the net positive charge. Peaks 5 and 6 were assigned to truncated forms that lost amino acids from the N-terminal side: M18-G103, N14-G103, Y13-G103 and S48-G103. In this case, the longer migration times compared to native TB10.4 might be the consequence of the loss of one (D5) or four (D5, D24, E38, E42) acidic amino acids. As indicated previously [8], these truncated species might result from aspecific protein digestion due to residual enterokinase activity.

**Table 1:** Species observed in BPEs of TB10.4 which has been exposed to glycosylation conditions (peaks 0–6) and TB10.4 which has been glycosylated with Man(1–6)Man-IME (peaks 0, 2, 3, 7–14)

Peak	Migration time (min)	Experimental mass (Da)	Assignment	Theoretical mass (Da)
0	20.6	11,076.1	TB10.4	11,076.3
1	17.9	9712.5	NA	–
		9510.4	NA	–
2	18.7	9296.2	A1-N86	9296.2
		9397.3	A1-T87	9397.3
		9528.4	A1-M88	9528.5
		9599.5	A1-A89	9599.6
		9730.6	A1-M90	9730.8
		9861.6	A1-M91	9862.0
		9932.7	A1-A92	9933.1
3	19.2	10,505.8	A1-E97	10,505.6
		10,647.9	A1-A99	10,647.8
4	20.2	11,092.1	Oxidized TB10.4	11,092.3
5	21.0	9191.3	M18-G103	9191.1
		9636.5	N14-G103	9636.6
		9799.5	Y13-G103	9799.7
6	21.4	6234.8	S48-G103	6234.8
*7*	*18.5*	*9996.6*	*A1-A89-ManMan*	*9996.7*
		*10,127.6*	*A1-M90-ManMan*	*10,127.9*
		*10,258.7*	*A1-M91-ManMan*	*10,259.1*
		*10,329.8*	*A1-A92-ManMan*	*10,330.2*
*8*	*18.7*	*11,871.3*	*Deamidated TB10.4-(ManMan)*_*2*_	*11,871.5*
		*11,887.3*	*Deamidated oxidized TB10.4-(ManMan)*_*2*_	*11,887.5*
*9*	*19.2*	*11,474.2*	*Deamidated TB10.4-ManMan*	*11,474.3*
*10*	*19.8*	*11,870.4*	*TB10.4-(ManMan)*_*2*_	*11,870.5*
		*11,886.3*	*Oxidized TB10.4-(ManMan)*_*2*_	*11,886.5*
*11*	*20.4*	*11,473.3*	*TB10.4-ManMan*	*11,473.4*
		*11,489.2*	*Oxidized TB10.4-ManMan*	*11,489.4*
*12*	*20.7*	*10,033.6*	*N14-G10-ManMan*	*10,033.7*
		*10,196.7*	*Y13-G103-ManMan*	*10,196.8*
*13*	*20.9*	*9344.0*	*G20-G103-ManMan*	*9343.8*
		*9588.4*	*M18-G103-ManMan*	*9588.2*
*14*	*21.1*	*6632.0*	*S48-G103-ManMan*	*6631.9*

Species observed only in the glycosylated sample in italics

*NA* not assigned

The base peak electropherogram (BPE) also shows a component at 20.2 min (Fig. 1, peak 4), which is not well resolved from TB10.4. The mass observed for this component is 16 Da higher than the mass of TB10.4, suggesting an oxidation product. Oxidation induces a relatively small change in protein mass (+16 Da), but no change in the protein charge, resulting in a small decrease in migration time only.

#### Ag85B

**Fig. 2:**
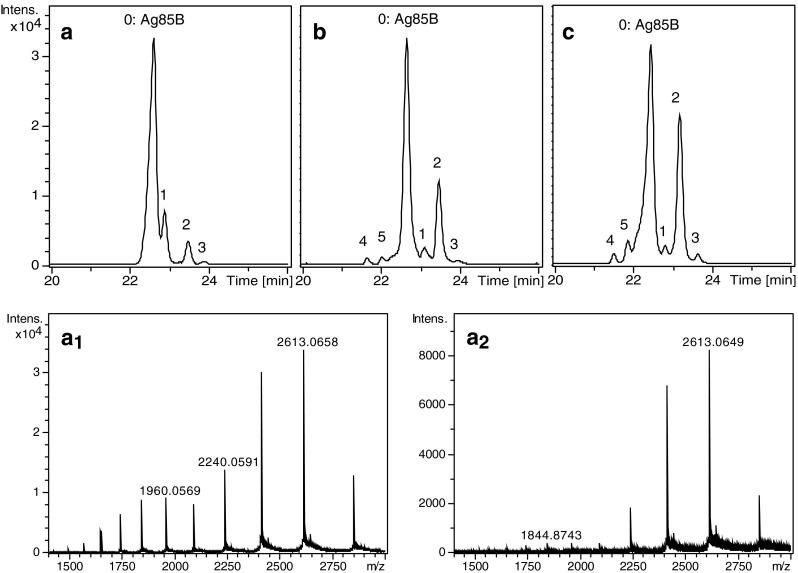
BPEs obtained during CE-MS of Ag85B (**a**) and Ag85B which has been exposed to glycosylation conditions for 24 h at 25 °C (**b**) or at 37 °C (**c**). Mass spectra obtained at the apex of peaks 0 (**a**_**1**_) and 1 (**a**_**2**_) of BPE of Ag85B (**a**)

CE-MS analysis of Ag85B in water showed the presence of four different species (Fig. 2a). The mass spectrum obtained for the main peak at approximately 22.5 min (peak 0) revealed, after deconvolution, a protein molecular mass of 31,345.7 Da, corresponding to the expected molecular weight of Ag85B (31,345.6 Da; see ESM, Fig. S4 for the protein sequence and Fig. S5 for the deconvoluted spectrum). The deconvoluted mass spectrum of the first component (peak 1) migrating after the main peak indicated the same mass as Ag85B, but the recorded mass spectra showed a clearly different charge state distribution (cf. Fig. 2a_1_, a_2_). The mass spectrum of the main peak (Fig. 2a_1_) seems to comprise two charge state distributions, which might be explained by partial unfolding of the protein during ESI, leading to increased charging and a shift of the distribution to lower *m*/*z* values. The mass spectrum corresponding to peak 1 (Fig. 2a_2_) shows a significantly lower relative abundance of the charge states at lower *m*/*z* values, indicating less susceptibility to unfolding. Peak 1 might be caused by a conformer of Ag85B exhibiting a different electrophoretic mobility and ESI.

Deconvolution of the mass spectrum obtained for peak 2 revealed a molecular weight of 30,424.3 Da, which was assigned to the truncated form R10-G292. The mass spectrum of low-abundant peak 3 indicates the same molecular mass as peak 2. However, the mass spectra of peaks 2 and 3 differ in their charge state distributions in a similar fashion as with peaks 0 and 1, again suggesting the presence of two protein conformers.

The Ag85B sample was exposed for 24 h to glycosylation conditions at both 25 and 37 °C. CE-MS analysis (Fig. 2b, c) showed the appearance of two additional degradation products (peaks 4 and 5), while the abundance of peaks 2 and 3 increased with respect to non-exposed Ag85B. Table 2 summarizes the peak assignment. The considerable increase of peak 2 is mainly caused by the formation of a new degradation product identified as the truncated form S9-G292, which co-migrates with R10-G292. Moreover, the abundance of R10-G292 relative to Ag85B slightly increased from 8.6 % in the non-exposed sample to 11.1 % in the sample incubated at 37 °C. The compound migrating as peak 5 at 21.9 min differs from Ag85B by +0.9 Da in mass only. Most probably, this modification relates to a single deamidation of the protein. Deamidation is a common protein degradation that results in a small change of protein molecular mass (+0.984 Da) and a shift of the p*I* due to the transformation of an amide residue into an acidic residue. The relative abundance of the degradation products was significantly lower at 25 °C. Therefore, this temperature was selected for Ag85B glycoconjugate synthesis.

**Table 2:** Species observed during CE-MS of Ag85B (peaks 0–3), Ag85B which has been exposed to glycosylation conditions for 24 h at 25 and 37 °C (peaks 0–5) and Ag85B which has been glycosylated with Man-IME (peaks 6 and 7)

Peak	Migration time (min)	Experimental mass (Da)	Assignment	Theoretical mass (Da)
0	22.5	31,345.7	Ag85B	31,345.6
1	22.8	31,345.6	Ag85B conformer	31,345.6
2	23.2	30,425.4	R10-G292	30,425.5
		30,512.4^a^	S9-G292	30,512.6
3	23.6	30,425.4	R10-G292 conformer	30,425.5
		30,512.4^a^	S9-G292 conformer	30,512.6
4	21.5	31,142.7^a^	A5-G285	31,143.3
5	21.9	31,346.8^a^	Deamidated Ag85B	31,346.6
*6*	*21.2*	*32,051.2*	*Ag85B-(Man)*_*3*_	*32,050.8*
		*32,286.2*	*Ag85B-(Man)*_*4*_	*32,285.8*
		*32,521.2*	*Ag85B-(Man)*_*5*_	*32,520.9*
		*32,756.2*	*Ag85B-(Man)*_*6*_	*32,755.9*
		*32,991.3*	*Ag85B-(Man)*_*7*_	*32,991.0*
		*33,226.1*	*Ag85B-(Man)*_*8*_	*33,226.0*
*7*	*21.7*	*31,453.2*	*S9-G292-(Man)*_*4*_	*31,452.8*
		*31,688.2*	*S9-G292-(Man)*_*5*_	*31,687.9*
		*31,922.9*	*S9-G292-(Man)*_*6*_	*31,923.0*
		*32,157.6*	*S9-G292-(Man)*_*7*_	*32,158.0*

Species observed only in the glycosylated sample in italics

^a^Detected only in BPEs of Ag85B which has been exposed to glycosylation conditions

### Characterization of TB10.4 and Ag85B glycoconjugates

TB10.4 and Ag85B were conjugated with Man and Man(1–6)Man activated as IME-glycosides according to the protocol previously reported (see “Materials and methods” and Fig. S6 in ESM for the reaction scheme). The resulting glycoconjugates were analysed with the optimized CE-MS method.

#### TB10.4 glycoconjugates

**Fig. 3:**
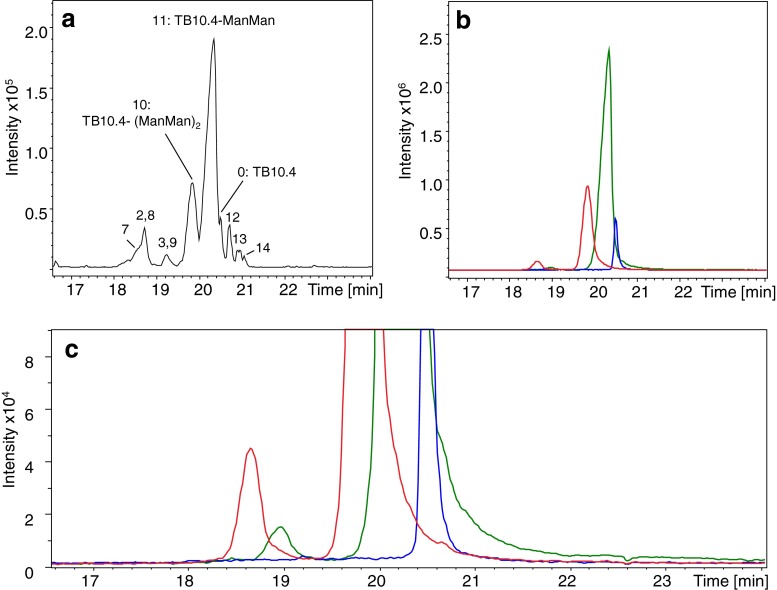
BPE (**a**) and EIEs (**b**) of most abundant ions of TB10.4 (*blue*), monoglycosylated TB10.4 (*green*) and diglycosylated TB10.4 (*red*) obtained during CE-MS of TB10.4 conjugated with Man(1–6)Man. **c** zoom of **b**

CE-MS analysis of TB10.4 conjugated with Man-IME and Man(1–6)Man-IME showed the presence of several components. The BPE obtained for TB10.4 conjugated with Man(1–6)Man-IME is shown in Fig. 3a; the mass spectra of the two main peaks are given in Figs. S7 and S8 in ESM. A number of additional peaks were observed in comparison to the unconjugated TB10.4 sample which was exposed to glycosylation conditions, but with no activated saccharide added (cf. Fig. 1). These peaks resulted mostly from the glycoconjugation of TB10.4. The main glycoconjugate species detected for the two samples had masses corresponding to TB10.4-Man and TB10.4-(Man)_2_, and TB10.4-Man(1–6)Man and TB10.4-[Man(1–6)Man]_2_, respectively. TB10.4 comprises one lysine residue as a potential glycosylation site, but as demonstrated in our previous work [17], under the applied glycosylation conditions, the amino functionality of the N-terminus of the protein also can be conjugated, leading to the formation of diglycosylated antigen. IME conjugation does not induce a change in the protein charge (see Fig. S2 in ESM), resulting in a partial CE separation of the unmodified protein and the somewhat larger glycoforms. For TB10.4 conjugated with Man(1–6)Man-IME, a better glycoconjugate resolution was reached due to the larger increase in antigen size (+397 Da with respect to +235 Da of Man-IME) upon conjugation (Fig. 3b).

**Fig. 4:**
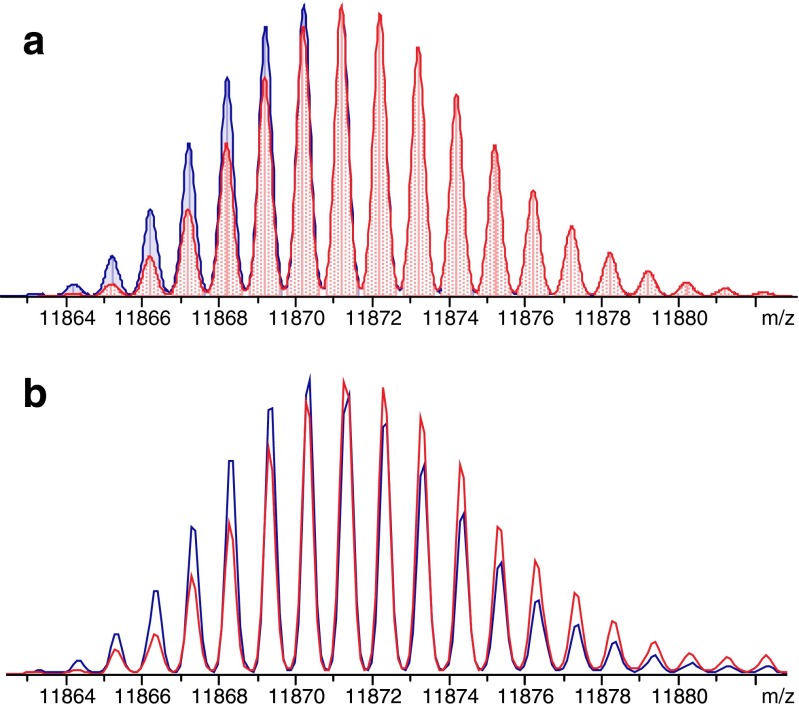
Deconvoluted mass spectra showing isotopic pattern of TB10.4-[Man(1–6)Man]_2_ (*blue*) and deamidated TB10.4-[Man(1–6)Man]_2_ (*red*) obtained **a** in silico and **b** during CE-MS

The truncated species identified in the antigen stability studies were also detected in the conjugated samples. The truncated forms that lost a lysine residue (peaks 2 and 3 in Table 1) with respect to native TB10.4 were detected both unmodified and glycosylated (at the N-terminal residue). The degradation products that still contained the lysine residue were mainly observed as glycosylated forms (Table 1). Interestingly, samples of TB10.4 conjugated with Man-IME as well as with Man(1–6)Man-IME showed two additional degradation products which were not observed during the stability studies (“Characterization and stability evaluation of TB10.4 and Ag85B”). These two compounds appeared to have molecular weights differing 0.9–1.0 Da from the mono- and diglycosylated TB10.4, respectively, and were detected at shorter migration times (Fig. 3c). Based on the molecular mass difference and migration time, we suppose the degradation products to be deamidated forms of the glycoconjugates. Partial C=C reduction and partial S–S cleavage could cause a similar isotope shift, but only when the reduction/cleavage product is not separated from the parent compound. However, the degradation products are separated from the parent compound and therefore most likely they are deamidated products. In order to test this hypothesis, the deconvoluted mass spectra of TB10.4-[Man(1–6)Man]_2_ and deamidated TB10.4-[Man(1–6)Man]_2_ were in silico simulated (Fig. 4a) and compared with the experimentally obtained deconvoluted mass spectra (Fig. 4b). The striking correspondence between the simulated and the experimental data, both showing a 1-Da shift of the isotopic distribution for the deamidated form in comparison with the non-deamidated one, strongly supports our assumption that deamidation occurred. As can be seen in Fig. 3, no deamidated form was detected for the unconjugated form of TB10.4. This result is in agreement with the stability study (“TB10.4”) where no deamidated species were observed for TB10.4. Assuming equimolar ESI efficiencies for the deamidated and non-deamidated glycoconjugates, deamidation ratios were estimated (Table 3). Higher rates of deamidation were obtained for the diglycosylated species (7.1–7.3 %) in comparison with the monoglycosylated ones (0.5–0.6 %). All together, these semi-quantitative results indicate a correlation between deamidation incidence and degree of glycosylation.

**Table 3:** Relative abundance of deamidated species observed during CE-MS of TB10.4 glycosylated with Man-IME and Man(1–6)Man-IME

Protein	Ratio deamidated/non-deamidated (%)
TB10.4	0.0
TB10.4-Man	0.5
TB10.4-ManMan	0.6
TB10.4-(Man)_2_	7.1
TB10.4-(ManMan)_2_	7.3

#### Characterization of Ag85B glycoconjugates

**Fig. 5:**
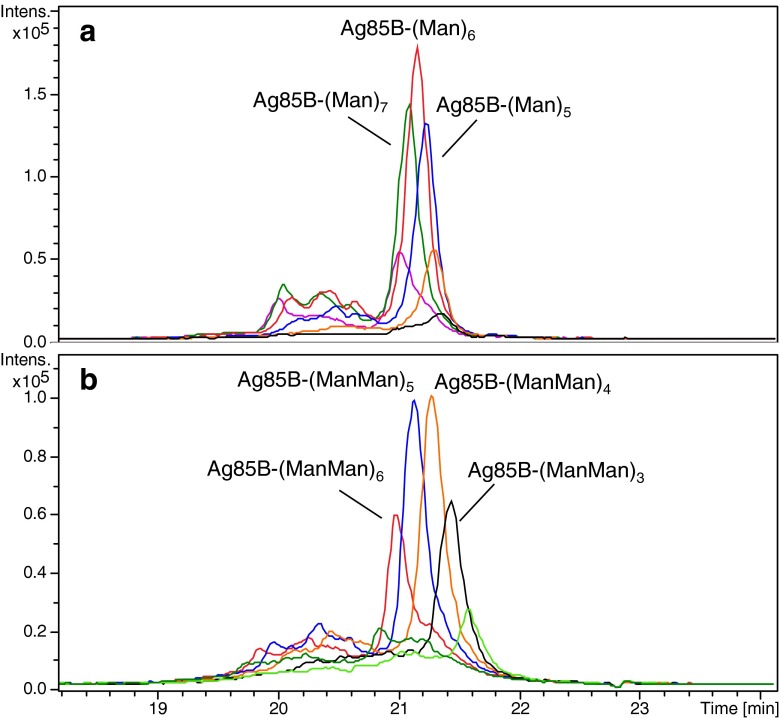
EIEs of most abundant ions of Ag85B conjugated with 2 (*light green*), 3 (*black*), 4 (*orange*), 5 (*blue*), 6 (*red*), 7 (*dark green*) and 8 (*purple*) glycan units obtained during CE-MS of Ag85B conjugated with **a** Man and **b** Man(1–6)Man

Ag85B has eight lysine residues in the amino acid sequence as potential glycosylation sites. CE-MS analysis of Ag85B conjugated with Man-IME or with Man(1–6)Man-IME revealed the presence of six glycoforms in both samples. Based on the molecular weights indicated by the deconvoluted mass spectra (see Figs. S9 and S10 in ESM), these Ag85B glycoconjugates appeared to contain three to eight saccharide units for the Man sample and two to seven for the Man(1–6)Man sample. Summed extraction of the three most abundant ions for each glycoform generated the EIEs reported in Fig. 5. The most glycosylated species migrated faster. As with TB10.4, higher resolution was obtained for the Man(1–6)Man glycoforms. The broad and non-Gaussian shape of the observed peaks, as appeared more evident for the Man(1–6)Man sample, is most probably related to the overlap of different *neo*-glycoprotein isomers characterized by the same saccharide loading number but a different location in the amino acid sequence. Higher conjugation efficiency was observed for the Man with an average of 5.9 saccharide units per protein whereas only an average of 4.6 was obtained in the case of Man(1–6)Man.

Regarding antigen degradation (see “Ag85B”), only the most abundant degradation product (S9-G292; peak 2 in Fig. 2 and Table 2) was detected with low intensity as a mixture of glycoconjugates (Table 2 for Ag85B conjugated with Man-IME). EIE traces of Ag85B conjugated with Man-IME and Man(1–6)Man-IME (Fig. 5a, b) show the presence of a cluster of broadened peaks at lower migration times comprising glycoform ions with similar *m*/*z* values (±0.5). These peaks most probably correspond to a number of glycoforms differing 1 Da, indicating the occurrence of deamidation. Different peaks were observed in each trace suggesting the occurrence of multiple deamidations in the same protein. Due to the broadened peak shapes and low intensity, it was not possible to estimate the percentage of deamidation of each glycoform. Still, the EIEs in Fig. 5 indicate that glycoforms with a higher number of conjugated saccharides show a greater susceptibility for deamidation, as was also observed for TB10.4.

## Conclusion

The potential of CE-MS for the characterization of intact *neo*-glycoproteins prepared from two MTB antigens was demonstrated. The use of a non-covalently coated (PB-DS-PB) capillary in combination with an acidic BGE allowed efficient separation of different antigen proteoforms and degradation products. The coupling with high-resolution TOF-MS permitted mass assignment for most of the variants observed. The method was suitable for the characterization of protein antigens allowing to establish the occurrence of modifications at different stages of the glycovaccine production. For example, CE-MS revealed the presence of conformers in Ag85B. The method also permits evaluation of antigen integrity and stability under glycosylation conditions and showed multiple degradation products resulting from e.g. deamidation and truncation. For *neo*-glycoprotein samples, the efficiency of glycosylation, number of glycoforms and saccharide units can be assessed, also showing conjugation of degradation products. In addition, glycoform-specific deamidation was observed suggesting a relation between the number of saccharide units and the rate of protein deamidation.

The developed CE-TOF-MS method was found suitable for the assessment of the detailed composition of *neo*-glycoprotein samples. As such, this method represents a valuable tool for obtaining well-characterized products that will further drive the optimization of the *neo*-glycoprotein production process.

## Electronic supplementary material

Below is the link to the electronic supplementary material.

ESM 1 (PDF 343 kb)
