# Supplementary material for: Monitoring antigenic protein integrity during glycoconjugate vaccine synthesis using capillary electrophoresis-mass spectrometry
Source: Anal Bioanal Chem. 2016 Jul 2;408(22):6123–32. doi: 10.1007/s00216-016-9723-5 (PMC4981626; doi:10.1007/s00216-016-9723-5)
Supplement: Supplementary file 1 — (PDF 343 kb) [file 216_2016_9723_MOESM1_ESM.pdf]

Analytical and Bioanalytical Chemistry

Electronic Supplementary Material

Monitoring antigenic protein integrity during glycoconjugate vaccine synthesis using capillary electrophoresis-mass spectrometry

Sara Tengattini, Elena Domínguez-Vega, Caterina Temporini, Marco Terreni, Govert W. Somsen

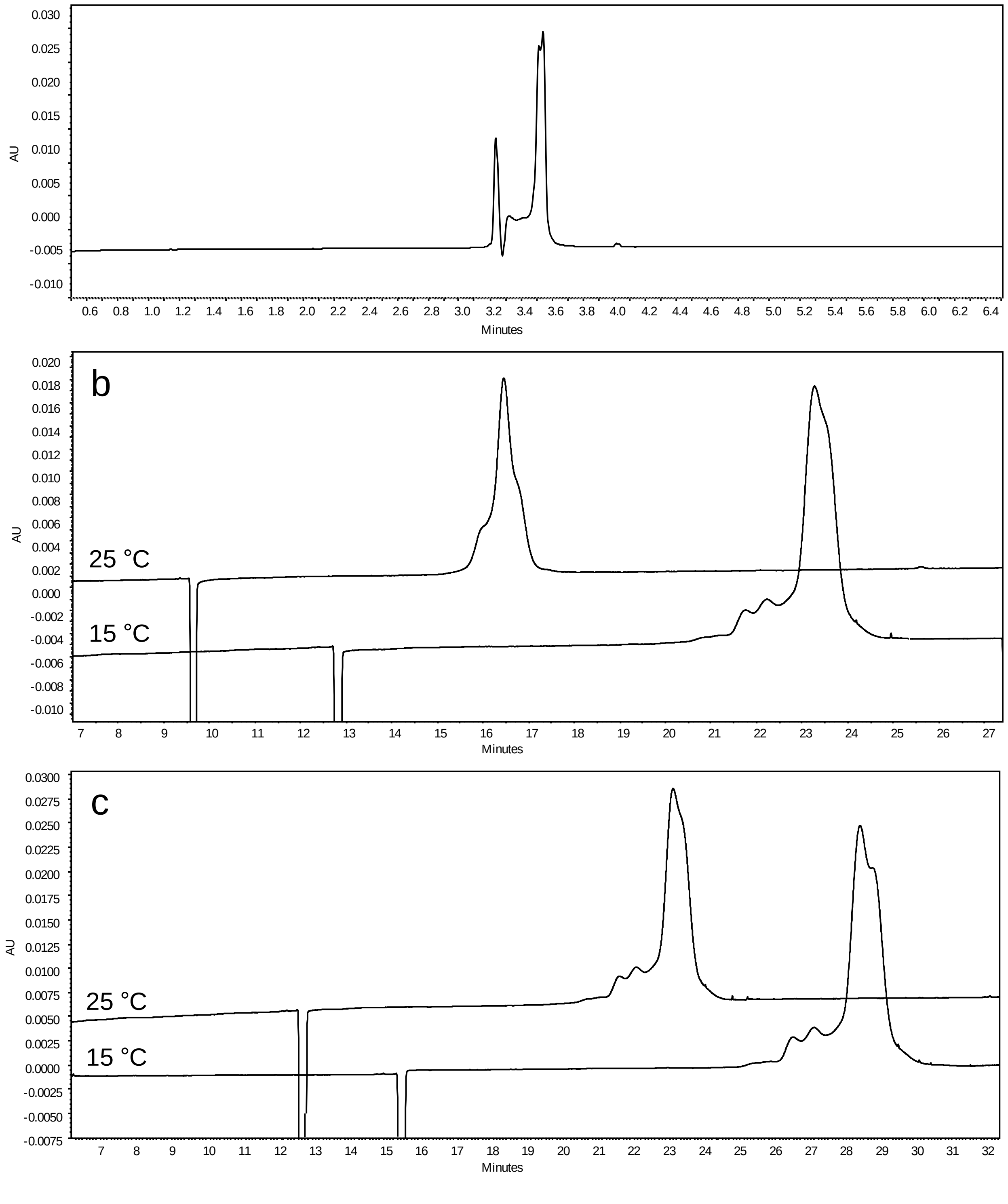

Fig. S1 CE-UV of Ag85B-Man using 200 mM (a), 1.5 M (b) and 2 M (c) acetic acid as BGE

TB10.4 (103 aa)

```
        10         20         30         40         50         60         70
AMAISDPMSQ IMYNYPAMLG HAGDMAGYAG TLQSLGAEIA VEQAALQSAW QGDTGITYQA WQAQWNQAME
        80         90        100
DLVRAYHAMS STHEANTMAM MARDTAEAAK WGG
```

Fig. S2 TB10.4 amino acid sequence. Lysine residue is coloured in red

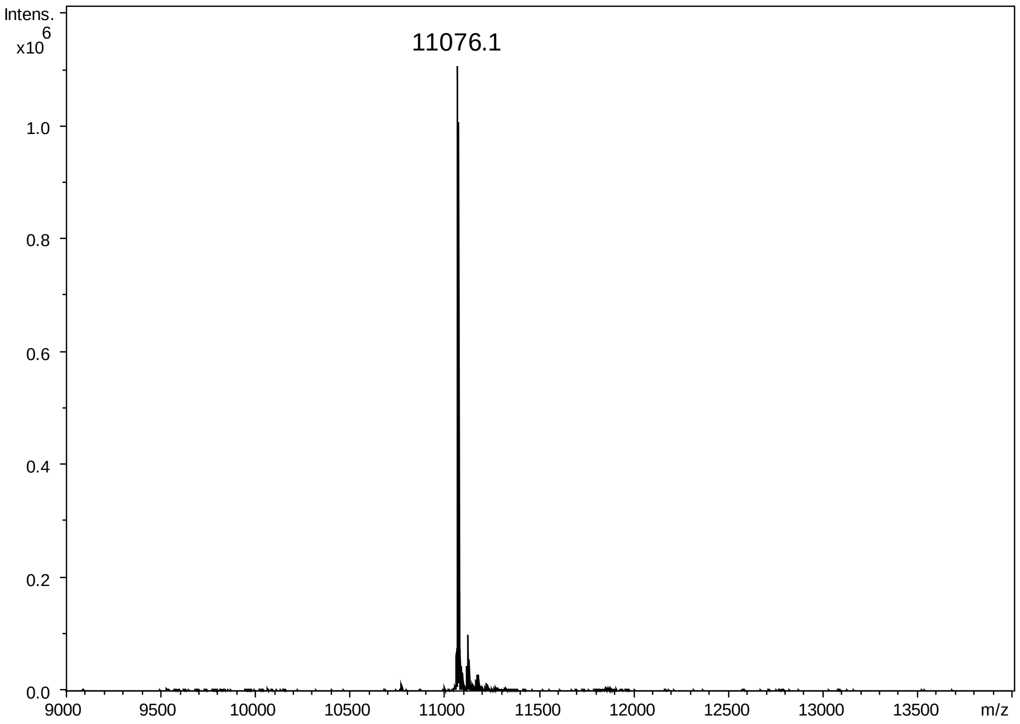

Fig. S3 Deconvoluted spectrum obtained for peak 0 in Fig.1. Mass of 11076.1 Da corresponds to TB10.4

Ag85B (292 aa)

```
        10         20         30         40         50         60         70
AMAISDPFSR PGLPVEYLQV PSPSMGRDIK VQFQSGGNNS PAVYLLDGLR AQDDYNGWDI NTPAFEWYYQ
        80         90        100        110        120        130        140
SGLSIVMPVG GQSSFYSDWY SPACGKAGCQ TYKWETFLTS ELPQWLSANR AVKPTGSAAI GLSMAGSSAM
       150        160        170        180        190        200        210
ILAAYHPQQF IYAGSLSALL DPSQGMGPSL IGLAMGDAGG YKAADMWGPS SDPAWERNDP TQQIPKLVAN
       220        230        240        250        260        270        280
NTRLWVYCGN GTPNELGGAN IPAEFLENFV RSSNLKFQDA YNAAGGHNAV FNFPPNGTHS WEYWGAQLNA
       290
MKGDLQSSLG AG
```

Fig. S4 Ag85B amino acid sequence. Lysine residues are coloured in red

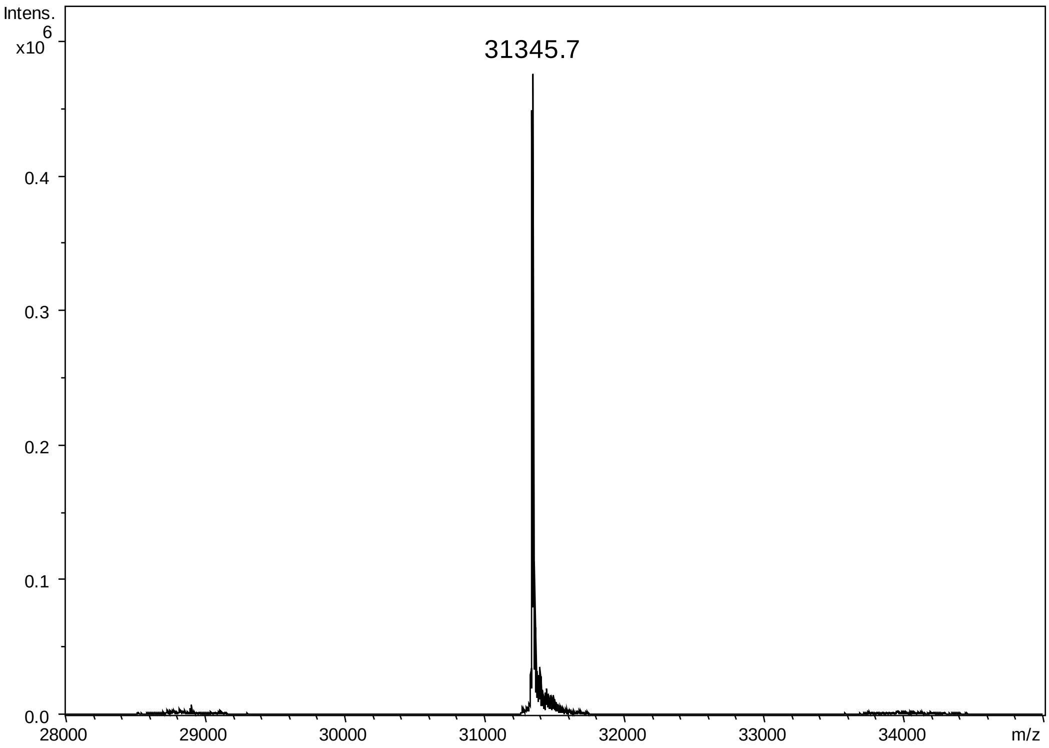

Fig. S5 Deconvoluted spectrum obtained for peak 0 in Fig.2a. Mass of 31345.7 Da corresponds to Ag85B

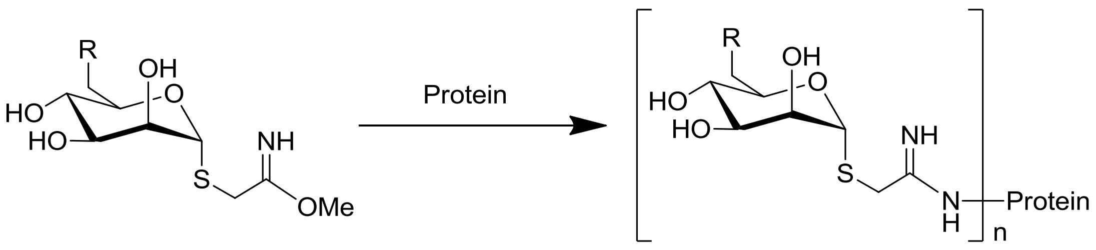

Fig. S6 General scheme of glycosylation reaction with IME-activated saccharides

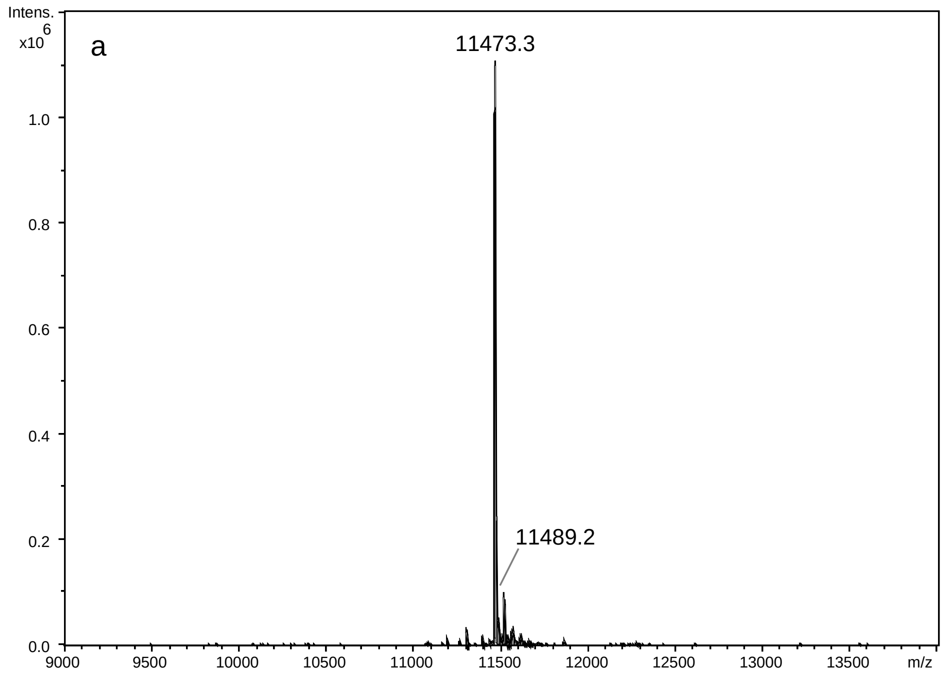

Fig. S7 Deconvoluted spectrum obtained for peak 11 in Fig. 3. Mass of 11473.3 Da corresponds to TB10.4-ManMan and mass of 11489.2 to oxidised TB10.4-ManMan

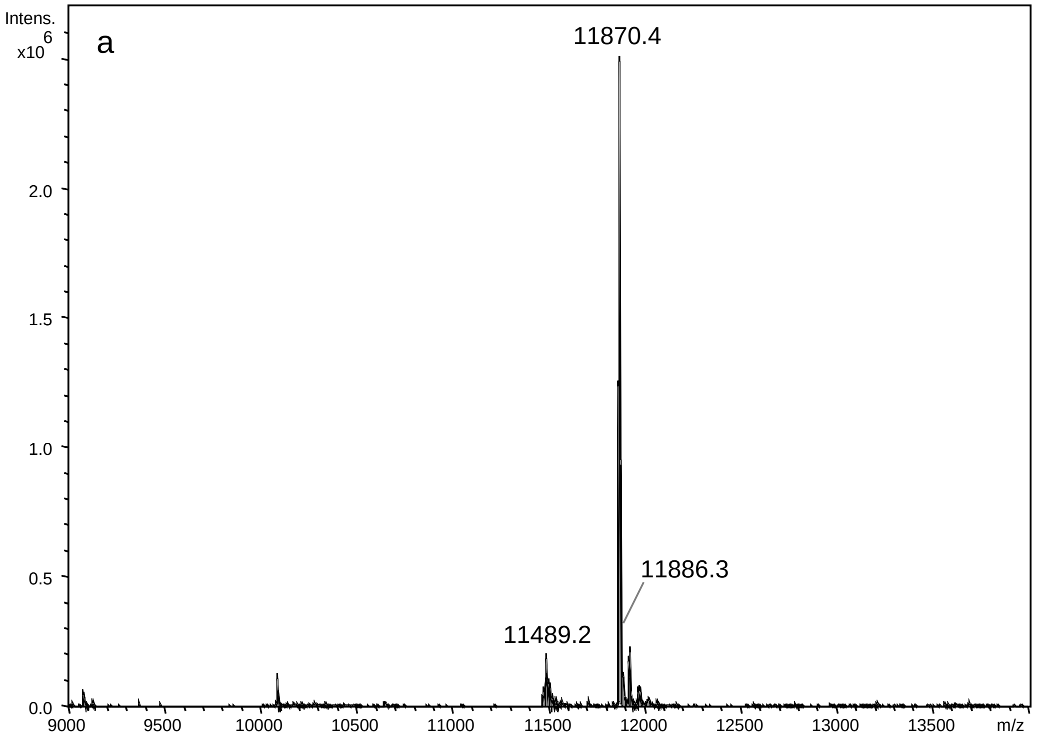

Fig. S8 Deconvoluted spectrum obtained for peak 10 in Fig. 3. Mass of 11870.4 Da corresponds to TB10.4-(ManMan)$_2$, mass of 11886.3 to oxidised TB10.4-(ManMan)$_2$

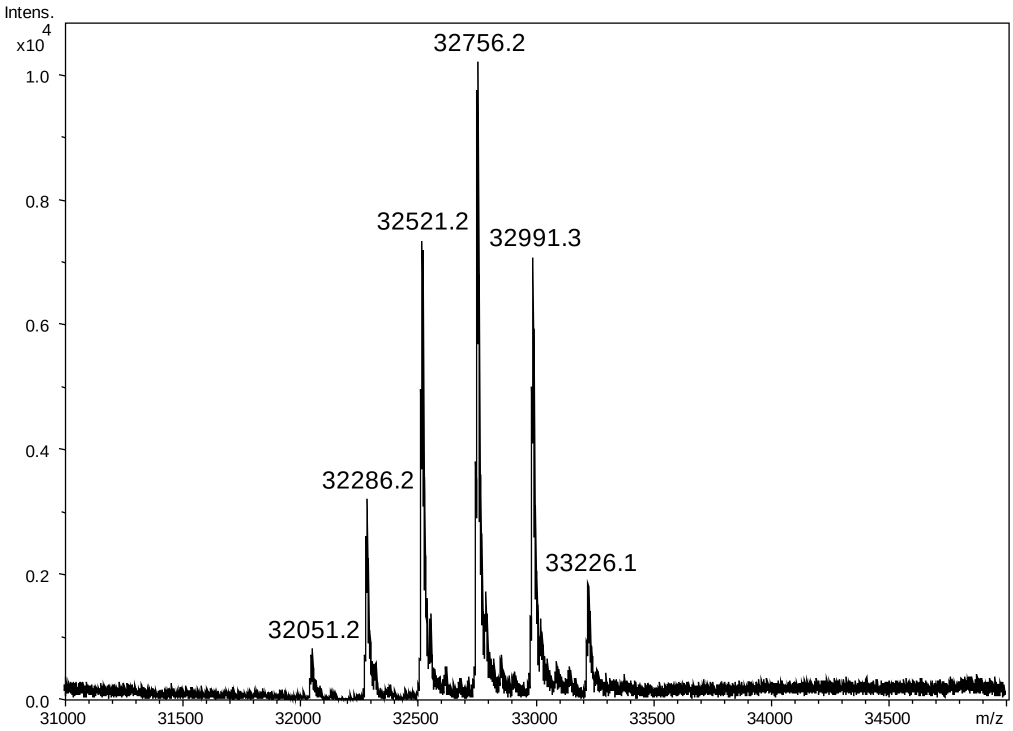

Fig. S9 Deconvoluted spectrum obtained between 20.8 and 21.4 min in Fig.5a. Masses of 32051.2, 32286.2, 32521.2, 32756.2, 32991.3 and 33226.1 Da correspond to Ag85B carrying from 3 to 8 Man units

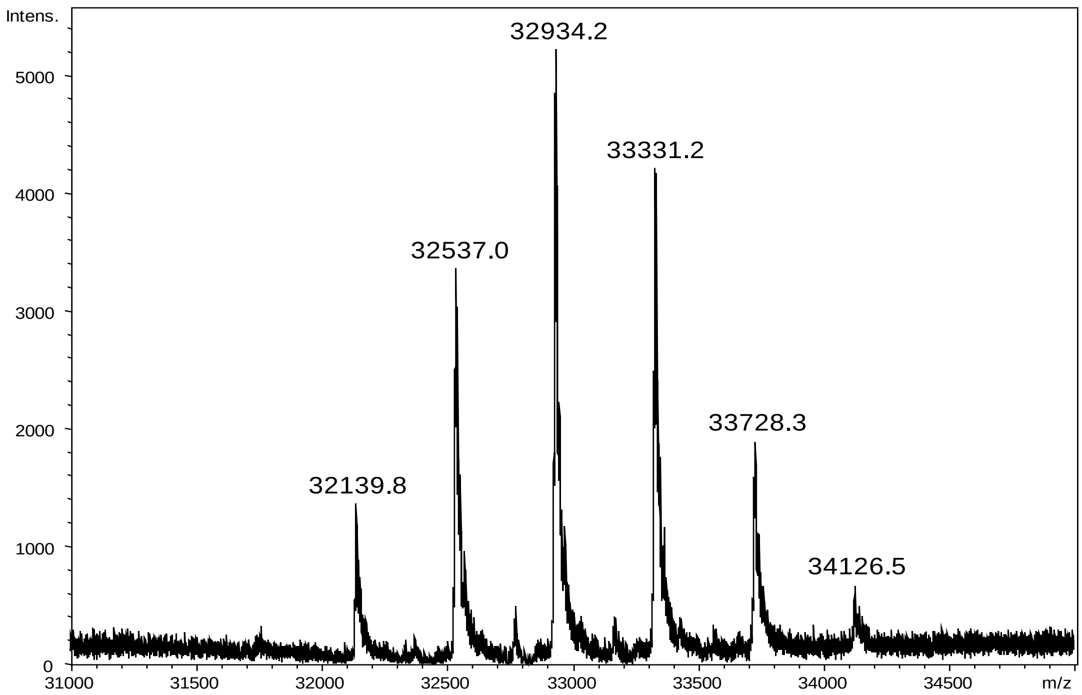

Fig. S10 Deconvoluted spectrum obtained between 20.8 and 22 min in Fig.5b. Masses of 32139.8, 32537.0, 32934.2, 33331.2, 33728.3 and 34126.5 Da correspond to Ag85B carrying from 2 to 7 Man(1-6)Man units
